# Preparation of a Breadfruit Flour Bar

**DOI:** 10.3390/foods5020037

**Published:** 2016-05-20

**Authors:** Carmen L. Nochera, Diane Ragone

**Affiliations:** 1 Department of Biomedical Sciences, Grand Valley State University, Allendale, MI 49401, USA; 2 Breadfruit Institute, National Tropical Garden, Kauai, HI 96741, USA; ragone@ntbg.org

**Keywords:** Breadfruit (*Meinpadahk*), gluten free bar, underutilized crop

## Abstract

Breadfruit is a nutritious, high energy food with a low quantity of protein but excellent protein quality. It has the potential to be developed into desired products which will help increase its utilization and add value to the crop. The overall purposes of this investigation were to develop a portable, nutritious, ready-to-eat breadfruit product (bar), test the sensory qualities of the product, and evaluate the nutritional properties of the product. Flour made from the Micronesian variety, *Meinpadahk* (*Artocarpus altilis* × *Artocarpus mariannensis*), was utilized for the development of the breadfruit bar. Breadfruit is a rich source of fiber, vitamins such as vitamin C, minerals such as potassium, and phytochemicals such as flavonoids. Nutritional labeling indicates that the breadfruit bar is high in carbohydrates and low in fat, and sensory evaluation indicates that 81% of the panelists found the bar acceptable while 19% disliked the bar. The breadfruit bar can provide an appealing and inexpensive gluten-free food source based on locally available breadfruit.

## 1. Introduction

The main objective of this study has been to develop a nutritional food bar centered on flour made from the Micronesian variety of breadfruit, *Meinpadahk* (see Appendix A). Until now, there has been no available literature pertaining to this quest. However, breadfruit is a common staple in Micronesia and, as a replacement of wheat flour, potentially can increase food production in Micronesia and across the globe. This study illustrates the development and evaluation of this food product using breadfruit flour augmented by other nutritional substances, completely available in Micronesia.

Breadfruit has drawn attention because of its abundance throughout tropical regions, low cost and great versatility as a food source [1,2,3,4,5,6,7,8,9,10,11,12,13]. Breadfruit (*Artocarpus altilis, Artocarpus mariannensis*) is a tropical plant requiring a warm, humid climate and plenty of rainfall. Breadfruit is round or oval, 3 to 8 in (9–20 cm) long and weighs 2–10 pounds (1–5 kg). The breadfruit tree produces two main crops throughout the year and the pulp, which is usually eaten, surrounds the heart or core and is white or yellowish depending on the maturity [2,9,14,15,16]. Although the quantity of protein in breadfruit is low, its quality is excellent [5]. It contains a high percentage of carbohydrates, primarily starch [1,6]. Breadfruit is typically eaten at the mature starch stage and can only be eaten raw at the soft, sweet ripe stage [1,2,10,11,12,14,15,16,17]. It may be eaten boiled, baked, roasted, pickled, steamed or fried [1,2,3]. In developing tropical countries, there is great need to utilize local food crops. This is especially true for those that are produced abundantly, economically, and are well liked by the locals. Breadfruit meets these criteria and provides an excellent source of calories for the diet. In addition to carbohydrates, it is also a rich source of fiber, vitamins such as vitamin C, minerals such as potassium, and phytochemicals such as flavonoids [1,2,10,12,18]. An additional nutritional benefit is that it is gluten-free [2,18]. Breadfruit represents a valuable food resource; however, its current usage is limited by the poor storage properties of the fresh fruit [2,9,11,16,19,20]. Conversion to flour, which has been performed by several investigators, provides a more stable storage form [7]. Formulation of a breadfruit cereal has also been successful and improves its storage capabilities [8,21,22,23,24,25].

Development of a convenient, nutritious, ready-to-eat breadfruit product could provide a locally grown food source of acceptable taste and nutritional value. The proposed breadfruit product may contribute to the global solution of alleviating world hunger [26].

## 2. Materials and Methods

### 2.1. Harvest and Preparation of Breadfruit (Meinpadahk)

The Micronesian variety, *Meinpadahk* (*Artocarpus altilis* × *Artocarpus mariannensis*), was utilized for the development of the breadfruit bar. Mature breadfruit was harvested by hand from trees in the McBryde Garden of the National Tropical Botanical Garden, Kalaheo, Kauai, Hawaii. Washed breadfruit was peeled, and the pulp was sectioned and dried at 80 °C for 24 h. Dried pulp was ground in a mill (waring) to produce flour that passed through an 80 mesh (180 µm) sieve.

### 2.2. Preparation of the Bar

The ingredients, dried papaya, honey, rice puffed cereal, cinnamon and vanilla, were purchased commercially. The ingredients were mixed thoroughly and stirred continuously in order to obtain a homogenous mixture. The batter was spread 1.27 cm thick on a cookie sheet and baked at 177 °C for 10 min. The batter was allowed to cool, and then cut into 2.5 cm by 7.5 cm bars. The recipe formulation is listed in Table 1.

### 2.3. Chemical and Nutritional Analyses of the Bar

Proximate analysis (crude fiber, ash, moisture) was performed on the breadfruit bar according to procedures outlined by Association of Official Analytical Chemists (AOAC), August, 2005 [27].

Nutrition labeling (calories, calories from total fat, total fat, fatty acids (saturated, trans and poly/mono unsaturated fat), cholesterol, sodium, total carbohydrate, dietary fiber, sugars, protein, vitamins A and C, calcium, iron) was performed according to procedures outlined by AOAC, Auguest, 2005 [27].

### 2.4. Sensory Evaluation of the Breadfruit Bar

The breadfruit bar was tested for acceptability of taste using a Hedonic test according to Larmond [28] and Meeilgard [29]. The product was evaluated by 62 volunteer semi-trained panelists. A nine-point verbal category hedonic scale was used: 1, dislike extremely; 5, neither like nor dislike; 9, like extremely. Data obtained from the taste panel was analyzed using the Z test for one proportion.

The study was approved by the Human Research Review Committee at Grand Valley State University, Allendale, Michigan. Informed consent was obtained from each participant. Exempt determination under category 45CFR 46.101 (b) (6) 14-160-H.

## 3. Results and Discussion

Results of label analyses based upon proximate analyses are presented in Table 2. Each 56 g bar provided 3.8 g of dietary fiber and 1.97 g/100 g of crude fiber.

Although the nutrition labeling results indicate this bar is low in fiber, there is variability among the reported fiber content of breadfruit [10,12]. This may be dependent upon species, maturity, processing, or type of analysis used for determination of fiber [12]. According to the USDA National Nutrient Database, fiber content for cooked potato, rice, sweet potato, taro, and plantains is 1.50, 0.30, 3.30, 5.10 and 2.30 g/100 g, respectively [30]. Turi *et al.* [12], and Ragone and Cavaletto [10] reported 100 g of cooked breadfruit can contain up to 7.37 g crude fiber. Rice is a commonly used ingredient in cereal bars. The addition of breadfruit flour to rice products can increase the fiber content of these products. Fiber has been demonstrated to reduce the incidence of degenerative diseases such as cancer, cardiovascular disease and diabetes [31].

The glycemic index (GI) reflects the degree to which a food raises the blood glucose [4,32]. Studies have demonstrated that cooked breadfruit has a low to moderate GI; hence, it can prevent hyperinsulinemia [12,32,33]. However, the addition of honey to the breadfruit bar for flavor and consistency increases the carbohydrate content and glycemic load. To date, there have been no published studies on the glycemic index of products developed from breadfruit flour [32].

Breadfruit is gluten-free [12,18], offering great potential in diversifying its uses in food product development for those who suffer from celiac disease and gluten allergies. The cost of gluten-free products is high. Breadfruit flour can be economical as a substitute for gluten flours [2].

In developing countries, the production of wheat is below domestic consumption. African countries, for example, import tremendous quantities of wheat [21,22,23,24,25]. If breadfruit can be used to substitute some of the wheat, this may help reduce expenditure of foreign exchange. In addition, high utilization of breadfruit will favor agricultural development, commerce, and availability of jobs within the indigenous nations [2,7,8,12,17,34,35,36,37].

The rapid increase in population in developing countries and the shortage of animal protein calls for urgent action in finding a suitable local crop which can be fortified with protein as economically as possible. The addition of high protein flour to the breadfruit mixture may be one solution [5].

There is no available literature pertaining to the development and evaluation of the nutritional value of a portable, ready-to-eat product produced from the Micronesian variety of breadfruit, *Meinpadahk*. This data is necessary if new products are to be developed in which breadfruit replaces wheat flour. With its great potential to increase food production, breadfruit may contribute to the global solution of alleviating world hunger.

**Figure 1:**
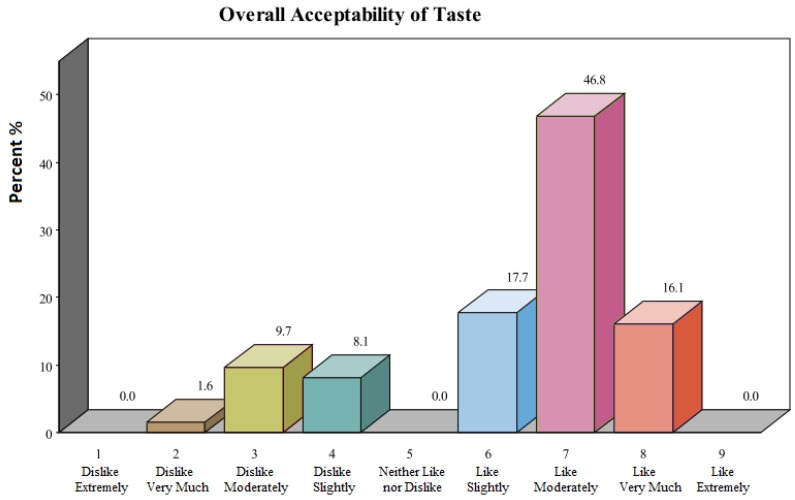
Overall acceptability of taste.

Sensory evaluation results are presented in Figure 1. Nineteen percent (19%) disliked the product, defined as a score of <5.0 on a nine-point hedonic scale. Liking the bar was defined as a score of six or greater. Eighty-one percent (81%) rated the bar as acceptable. A Z test for distribution demonstrated a probable range of 70%–90% rating the bar as acceptable (95% Confidence Interval: 0.7076, 0.9044).

## 4. Conclusions

The overall purposes of this investigation were to develop a ready-to-eat food source based on locally available breadfruit in areas of the world where it can be easily grown, test the sensory qualities of the product, and evaluate is nutritional properties.

The study indicates that a bar can be produced utilizing breadfruit flour developed from the Micronesian variety, *Meinpadahk*. Sensory analyses demonstrate acceptability; therefore, the bar may compete with already existing bars in the target market. Breadfruit flour is moderate in fiber compared to grains traditionally used in portable bars. Breadfruit flour is also gluten-free, making it attractive for those with celiac disease and gluten insensitivity. Based on these findings, one can infer that a nutritious, portable, gluten-free bar made with breadfruit flour may be a possible solution to helping alleviate hunger in underdeveloped areas, where the crop is indigenous. The bar is not an energy or protein bar and is not intended for weight loss. There is great need to expand the cultivation of breadfruit throughout these regions, enhancing its utilization and market potential.

## Figures and Tables

**Table 1:** Breadfruit bar (*Meinpadahk*) ingredients.

Ingredients	Grams (g)	Source
Breadfruit flour	125	McBryde Garden, National Tropical Botanical Garden, Kauai, HI
Ground dried papaya	125	Harvest Foods, West Michigan
Honey	125	Harvest Foods, West Michigan
Puffed rice cereal	125	Kellogg Co., Battle Creek, MI
Cinnamon	2.5	Harvest Food, West Michigan
Vanilla	2.5	Harvest Foods, West Michigan

**Table 2:** Nutritional label analysis.

Analysis	Result per 100 g	Result per Serving Size 1 bar (56 g)	Label Declaration	% Daily Value
Calories	348	195	190	
Calories From Total Fat	4	2	0	
Total Fat	0.43 g	0.24 g	0 g	0
Saturated Fat	0.2 g	0.1 g	0 g	
Trans Fat	<0.1 g	<0.1 g	0 g	
* Polyunsaturated Fat	0.2 g	0.1 g	0 g	
* Monounsaturated Fat	0.1 g	<0.1 g	0 g	
Sodium	114 mg	64 mg	65 mg	3
Total Carbohydrate	83.92 g	47.00 g	47 g	16
Dietary Fiber	3.8 g	2.1 g	2 g	9
Sugars	42.72 g	23.92 g	24 g	
Protein	2.05 g	1.15 g	1 g	
Vitamin A	191 IU	107 IU	0.02	
Vitamin A	50 RE	28 RE		
Vitamin C	0.8 mg	0.4 mg	0	
Calcium	58 mg	32 mg	0.04	
Iron	1.99 mg	1.11 mg	0.06	
* Ash	1.10%			
* Moisture	12.50%			
* Crude Fiber	1.97%			

* = Non-mandatory or voluntary label declarations; IU = International Units; RE = Retinol Equivalents.

**Figure A:**
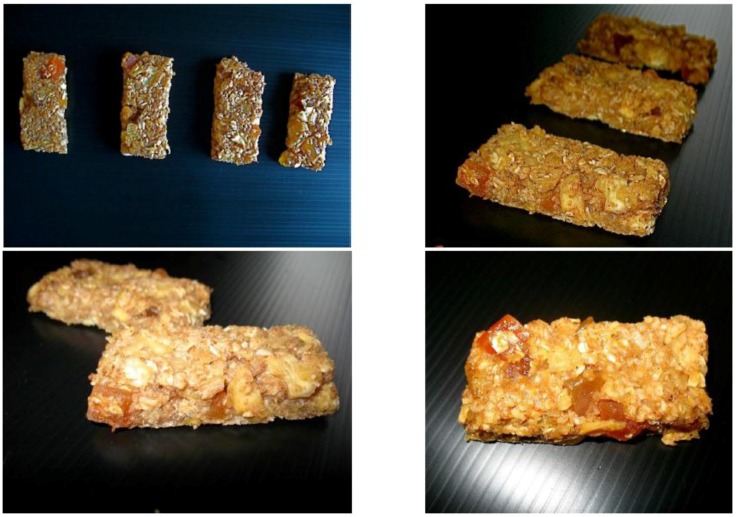
Breadfruit bar^©^ 2014 Mark Wilkens.
